# Deforestation and Carbon Loss in Southwest Amazonia: Impact of Brazil’s Revised Forest Code

**DOI:** 10.1007/s00267-017-0879-3

**Published:** 2017-05-16

**Authors:** Pedro Augusto Costa Roriz, Aurora Miho Yanai, Philip Martin Fearnside

**Affiliations:** 1 National Institute for Research in Amazonia (INPA), Av. André Araújo, 2936, Manaus, Amazonas CEP 69067-0375 Brazil; 2 Brazilian Research Network on Climate Change (RedeClima), Manaus, Brazil; 3 Instituto Federal de Educação, Ciência e Tecnologia do Amazonas (IFAM), Humaitá, Amazonas CEP 69800-000 Brazil

**Keywords:** Global warming, Tropical forest, Climate change, Land-use change, Forest policy, Landscape dynamics

## Abstract

**Abstract:**

In 2012 Brazil’s National Congress altered the country’s Forest Code, decreasing various environmental protections in the set of regulations governing forests. This suggests consequences in increased deforestation and emissions of greenhouse gases and in decreased protection of fragile ecosystems. To ascertain the effects, a simulation was run to the year 2025 for the municipality (county) of Boca do Acre, Amazonas state, Brazil. A baseline scenario considered historical behavior (which did not respect the Forest Code), while two scenarios considered full compliance with the old Forest Code (Law 4771/1965) and the current Code (Law 12,651/2012) regarding the protection of “areas of permanent preservation” (APPs) along the edges of watercourses. The models were parameterized from satellite imagery and simulated using Dinamica-EGO software. Deforestation actors and processes in the municipality were observed in loco in 2012. Carbon emissions and loss of forest by 2025 were computed in the three simulation scenarios. There was a 10% difference in the loss of carbon stock and of forest between the scenarios with the two versions of the Forest Code. The baseline scenario showed the highest loss of carbon stocks and the highest increase in annual emissions. The greatest damage was caused by not protecting wetlands and riparian zones.

**Graphical Abstract:**

## Introduction

### The Forest Code and Its Political Context

The Brazilian Forest Code, as amended in 2012 (Table 1), reduces the “APP” on the banks of watercourses and around their headwaters. The new code no longer requires protecting vegetation around intermittent streams, and it reduces the need for restoring this vegetation in properties where illegal deforestation of APPs took place before 22 July 2008 (Brazil 2012). The new rules that compute APP boundaries by measuring from the edges of watercourses eliminate protection of the floodplain (*várzea*) and, consequently, transitional environments between flooded and non-flooded ecosystems. These environments are of great importance to biogeochemical cycles and to the survival of various species (Piedade et al. 2012). When landscapes are heavily deforested, APPs along watercourses provide forest corridors connecting the remaining forest fragments, thus allowing movement of both animals and plants among fragments (de Marco and Coelho 2004). This is vital to maintaining biologically viable populations. Riparian vegetation reduces the amount of sediment deposited in rivers and the incidence of floods (Tundisi and Tundisi 2010).

**Table 1:** Comparison between the 1965 and 2012 Forest codes

Characteristic	1965 Forest code	2012 Forest code
Assumed relationship between forest and agriculture	Presence of forests is necessary for the maintenance of agricultural activities	Presence of forests is an obstacle to expansion of production
Assumed priority for increasing production	Production should increase through use of conservation techniques and better technologies	Production should increase as the available area increases
Dimensions of APPs along the edges of watercourses	Measure from the maximum water level	Measure from the “regular” water level
Requirement for restoration of illegally cleared APPs along the edges of watercourses	Requirement depends on the width of the watercourse	Requirement depends on the size of the property
APP included in the percentage of the legal reserve	Under certain special conditions	Always included
APP around non-perennial springs	Always required	Not required
APP in wetlands	Always required	Only required if declared as “of social interest”
Provision for compensating elsewhere for clearing in the legal reserve	Must be in the same watershed	Must be in the same biome^a^
Legal reserve restoration using exotic species	Only allowed in small family farming properties	Allowed in all properties
Maintenance/restoration of the legal reserve	All properties must maintain the legal reserve and restore it if cut	Properties with up to four tax modules need not restore it if cut by 2008

^a^ Since 2004 much of Brazil’s planning has been based on division of the country into six “biomes,” the “Amazonia biome” being the area where the original (pre-Columbian) vegetation was predominantly Amazonian forest, although it also includes enclaves of other vegetation types such as savannas (Brazil, IBGE 2004)

The justification for revising the Forest Code was to increase agricultural production and facilitate land “regularization” (Rebelo 2010). “Regularization” (*regularização*) means bringing the status of land into conformance with the law, for example by planting trees in areas deforested illegally, paying fines, or by simply pardoning past illegal deforestation. However, the revised Forest Code reduced the protection of ecosystems by reducing areas required for APPs. The argument that more land was needed to increase agricultural production has been challenged (Martinelli et al. 2010; Michalski et al. 2010). There appeared to be no ethical or economic reasons for updating the law (Vieira and Becker 2010), leaving the possibility of regularizing illegalities as the main motivation.

Reduction in protected areas, together with other measures to reduce environmental liabilities and to facilitate environmental “regularization,” can result in the reduction of native vegetation and increasing greenhouse-gas emissions. Revision of the Brazilian Forest Code was therefore a setback for global efforts to mitigate climate change (IPAM 2011; Martinelli 2011; Metzger et al. 2010; Soares-Filho et al. 2014).

The long debate over revising the Forest Code, and the votes in the House of Deputies and Federal Senate, represent a turning point in the political context of environmental regulation in Brazil that extends far beyond the effects of the changes in the Forest Code itself. The House of Deputies approved the revision in 2011 by a ratio of seven to one. Brazil’s population is 85% urban, meaning that the vast majority of voters have no financial interest in being allowed to deforest more, especially in high-risk areas like steep hillsides and near watercourses. During the debate itself dramatic reminders of the importance of forests in these areas were provided by over 200 deaths from landslides in the state of Rio de Janeiro and thousands of families displaced by overflowing rivers in northeastern Brazil. At the time of the vote, public opinion polls showed 80% of Brazil’s population opposing any change in the Forest Code (Lopes 2011). How, then, can one explain the members of the House of Deputies, where representation is proportional to population, voting overwhelmingly against the interests of their own constituents? The logical explanation lies in power of money from soy and other agribusiness interests being transformed into political influence (Fearnside and Figueiredo 2016). The most important result of the vote was its dramatic demonstration of the influence of the “ruralist” block (representatives of large landholders), both leading to appointment of ruralist leaders to many key positions in the government and to a shift in expectations of the public, especially in rural areas. The year 2012, when the Senate approved the revision and the new Forest Code entered into effect, coincides with a reversal of the downward trend in Brazil’s Amazonian deforestation rates that had been in course since a peak in 2004. The annual deforestation rate jumped by 29% in 2016; while several factors were pushing in this direction, the magnitude of the surge suggests that it also had roots in a further spectacular increase in the political power of the ruralists in that year (Fearnside 2017). Among the effects of this rise in influence is a series of proposed laws and constitutional amendments that would essentially dismantle Brazil’s environmental licensing system (Fearnside 2016).

### Simulation Models

Simulation models have not often been used in assessing the impact of policies that have already been implemented (He et al. 2013), but deforestation modeling makes it possible to project the transition rates of land-use and land-cover change. This is needed to support policies to combat deforestation and to improve understanding of the mechanisms and causative agents of deforestation (Lambin 1994). Spatial modeling of deforestation can be done using Dinamica-EGO software (Rodrigues et al. 2007; Soares-Filho et al. 2002), which is based on a cellular automata system with transition rules set in accordance with the characteristics of the surrounding cells (White and Engelen 2000). A limitation of cellular automata-based models for simulating the effect of policies is that the inferences they make are restricted to changes in land use and land cover (He et al. 2013). Despite this restriction, Dinamica-EGO has been widely used to project deforestation (Soares-Filho et al. 2006) and to analyze the impacts of building roads (Barni et al. 2015; Fearnside et al. 2009; Soares-Filho et al. 2004) and the effect of policies for establishing conservation units (CUs) (Yanai et al. 2012). “CUs” are protected areas created under the National System of Conservation Units (SNUC) (Brazil, MMA 2015). “Protected areas” include both CUs and indigenous lands (ILs).

### Study Objectives

The aim of the present study was to quantify potential impacts on deforestation resulting from the changes in the Brazilian Forest Code and estimate the loss of carbon stock in the municipality of Boca do Acre, located in the southwestern portion of Brazilian Amazonia. Three scenarios were developed, and deforestation was simulated to 2025. A baseline scenario was simulated (considering the historical trend in deforestation rates in Boca do Acre) together with two scenarios whose premise is the total prohibition of deforestation within the APPs on the banks of watercourses as required by the two Forest Codes (1965 and 2012). The 1965 Forest Code measured the width of the APPs starting from the maximum water level in the watercourses, whereas the 2012 law begins the measurement from the “regular” channel, meaning the minimum water level. Especially in Amazonia, the edge of the “regular” channel is substantially lower than the previous starting mark, resulting in less area being protected. In addition to the APP reductions that are the focus of this study, the 2012 law reduces the area of forest protected by the “legal reserve” requirement (a percentage of each property that must be maintained as forest): although the required percentage remains the same, the APPs are now “incorporated” as part of the legal reserve rather than being additional to this requirement. The 2012 revision of Brazil’s Forest Code represents a major change affecting Amazonian forest, but most discussion of these impacts has been in general terms. Quantification of potential effects in a specific location adds an important dimension to this discussion.

## Materials and Methods

### Study Area

**Fig. 1:**
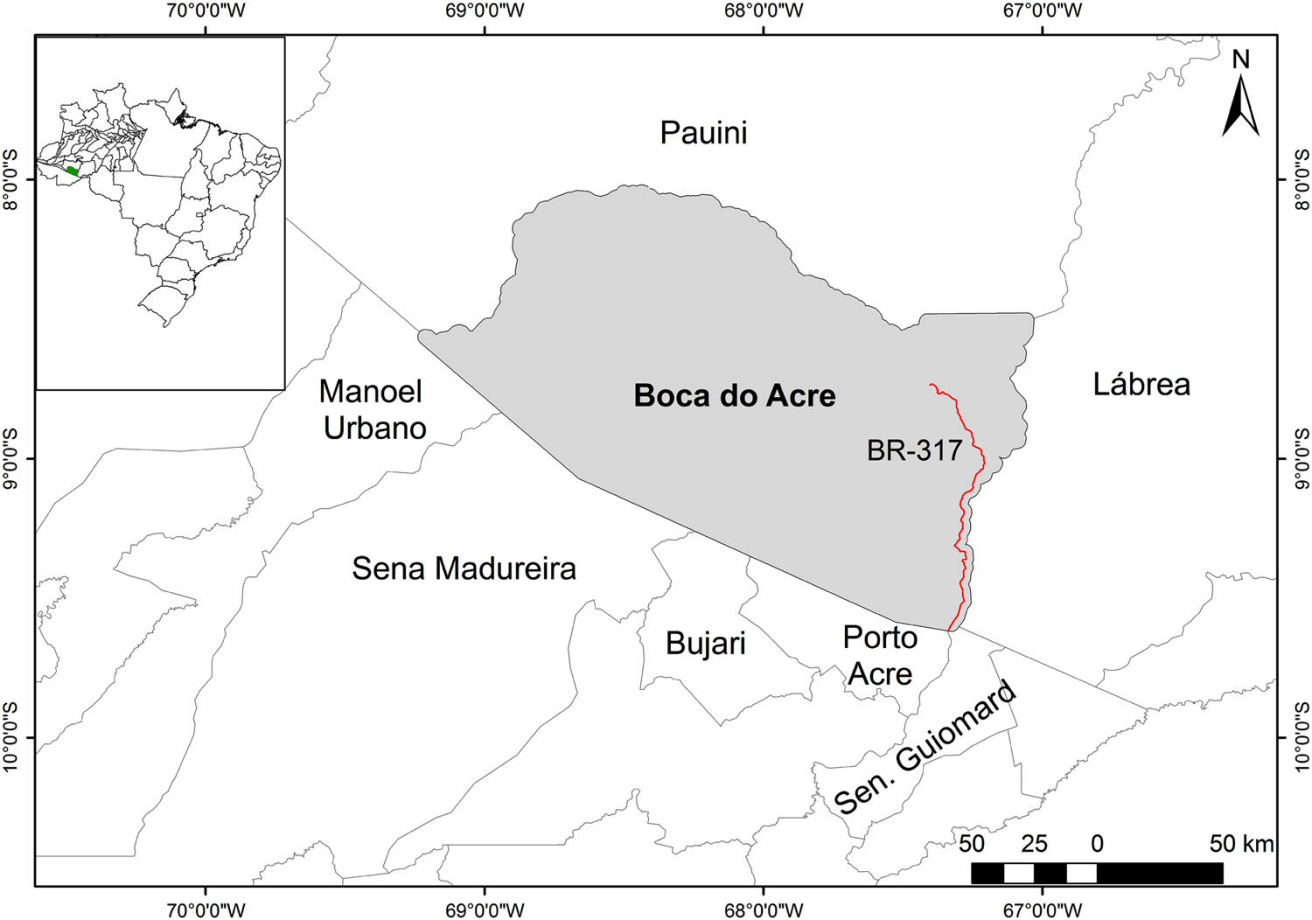
Study area

The study area comprised the municipality (county) of Boca do Acre in the state of Amazonas (located in the southwestern portion of Brazilian Amazonia). A 3-km buffer around the municipality was also included in order to capture the maximum effect of the BR-317 (Rio Branco—Boca do Acre) Highway and avoid interference from edges in modeling. The study area covered a total of 24,133 km^2^, including the buffer that encompassed small portions of the neighboring municipalities of Lábrea and Pauini in the state of Amazonas and Manoel Urbano, Sena Madureira, Bujari, Porto Acre, and Senador Guiomard in the state of Acre (Fig. 1).

Boca do Acre comprises an area 21,951 km² and has annual average rainfall of 2000 to 2400 mm (Sombroek 2001); its economic base is cattle, which, with 84,954 head, is the fifth largest herd among the 62 municipalities in the state of Amazonas (Brazil, IBGE 2011). The boundary of the municipality to the east follows Highway BR-317, which is an important factor increasing its attractiveness for deforestation (Piontekowski et al. 2011).

Deforestation through 2012 in Boca do Acre totaled 2076 km² (9%), which was the second highest deforestation extent in the state of Amazonas. The annual increase of 54.9 km^2^ (0.24% of the municipality) in 2012 was the largest in the state (Brazil, INPE 2016). Amazonas is Brazil’s largest state with 1.57 million km^2^, an area approximately the size of the US state of Alaska and more than double that of the state of Texas.

### Fieldwork

Fieldwork was carried out between 11 and 19 August 2012. Both small and large properties were visited to better understand the dynamics of land-use and land-cover change in the municipality. The routes of roads that were not included in official maps were collected using a GPS, and the widths of the main rivers at their maximum water levels were observed to supply information needed by the models.

### Acquisition and Processing of Images

Imagery from the thematic mapper sensor on the Landsat-5 satellite was used (30-m spatial resolution) for the years 2005, 2008, and 2010. The images were obtained from the National Institute for Space Research (INPE) (http://www.dgi.inpe.br/CDSR/) for the relevant rows and points (3/66, 2/66, 1/66, 1/67, and 2/67). For 2012 we used images from the LISS3 sensor on the ResourceSat-1 satellite (23.5-m spatial resolution). These images were then resampled to 30 m. All images were georeferenced on the basis of the GeoCover 2000 mosaic of the US National Aeronautics and Space Administration (https://zulu.ssc.nasa.gov/mrsid/). The cartographic projection applied was UTM Zone 19 South and Datum WGS1984.

**Fig. 2:**
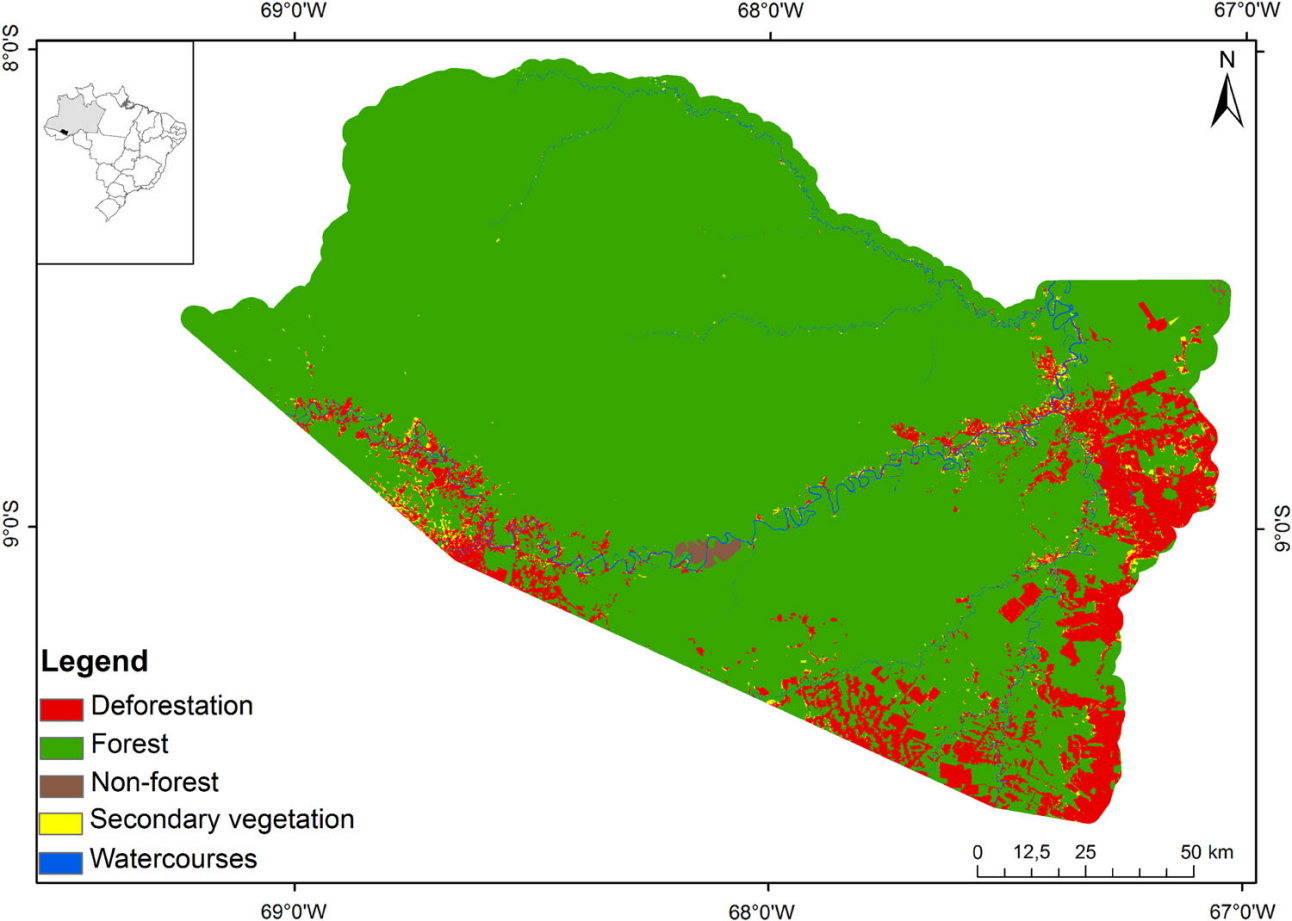
Land-cover map for 2012

The portion representing the study area was cut out of a mosaic created from georeferenced images. The clipped images were classified into forest, non-forest, watercourses, secondary vegetation, and deforestation according to the methodology proposed by Graça and Yanai (2008) using as a classifier the maximum similarity determined in ENVI software. The result of this process for 2012 is shown in Fig. 2.

### Delimitation of Hydrography and APPs

Watercourses and their associated APPs were divided into:

- Watercourses < 30 m in width;
- Watercourses ≥ 30 m in width computing the APP from the “regular” channel;
- Watercourses ≥ 30 m in width computing the APP from the maximum water level.

For rivers with width less than 30 m, SRTM (Shuttle Radar Topography Mission) images were used (available at http://www.relevobr.cnpm.embrapa.br/download/) with a spatial resolution of 90 m resampled to 30 m. From this procedure a digital elevation model was developed for the study area using “Arc Hydro Tools” in ArcGIS software, where the watercourses (hydrography) were bounded as demonstrated by Alves Sobrinho et al. (2010).

Watercourses ≥30 m in width, were extracted from classified images (Fig. 2) and the width of each river was measured using the “measure” tool in ArcGIS for determining the APPs, as demonstrated by Reich and Francelino (2012). To measure the width of the rivers at the maximum water level a mask of the flooded areas of the Amazon region was used with 100-m spatial resolution produced from the synthetic-aperture radar sensor on the Japanese Earth Resource Satellite 1 (Hess et al. 2003, 2012). This mask was resampled to 30 m and, later, the floodplain of each river was measured using the “measure” tool to determine the APPs. The images were then clipped based on the study area, the rivers were grouped into width classes and the buffers for the APPs were created. Since the maximum resolution of the images was 30 m, we used modeled 30-m buffers to represent the APPs for all rivers with <30 m width (Table 2).

**Table 2:** Buffers built for APPs based on Law 12,651/2012

Width of watercourse	APP based on Law 12,651/2012	Buffer width
<30m	30 m (rivers up to 10 m in width) or 50 m (rivers 10 m to 50 m in width)	30 m
30 to 50 m	50 m	50 m
50 to 200 m	100 m	100 m
200 to 600 m	200 m	200 m
>600 m	500 m	500 m

Maps of APPs generated for the rivers with width ≥30 m were added separately to the map of narrow watercourses, producing two separate maps, one with the APPs computed from the “regular” channel (Law 12,651/2012) and the other with the APPs computed from the maximum water level (Law 4771/1965).

### The A-eco Model

The model used in the present study, denominated “A-eco,” was simplified from the AGROECO model that was created for simulating deforestation considering the influence of roads (IR) and the preservation offered by CUs (Fearnside et al. 2009). The main modifications refer to the simulated transition rate feedback and the inclusion of “regions” in the model.

In the AGROECO model transition rates were calculated in Vensim, which is a non-spatial simulation software (Ventana Systems Inc. 2007). In AGROECO, Vensim was coupled interactively with the 32-bit version of Dinamica-EGO, which performed the spatial allocation of the rates. In the A-eco model, transition rates were calculated using only the operators (“functors”) in the 64-bit version of Dinamica-EGO, thus no longer requiring the use of Vensim. This change was made because the coupling with Vensim hindered the use of maps with a large number of cells and because the 64-bit version of Dinamica-EGO is incompatible with Vensim. The 64-bit version of Dinamica-EGO allowed more-detailed *raster* maps to be used and has better performance in terms of processing time.

The approach consists of partitioning the “regions” into which we divided the study area so that processing is done separately for each of the “regions.” At the end of each iteration, the regions are grouped again into a single map. The iterations (repetitions of the model calculations) in this case represent years. In the present study, the regionalization of the study area allowed calculating the rates of deforestation, with projection of road construction specific to each region. This allowed capturing the particularities of deforestation for each agent or intrinsic focus of deforestation. In addition, using this approach it was possible to compute the transition rates for each region in order to construct the scenarios used for comparing forest loss and carbon emissions under different versions of the Forest Code and under different assumptions regarding enforcement.

### Input Variables in the A-eco Model

**Fig. 3:**
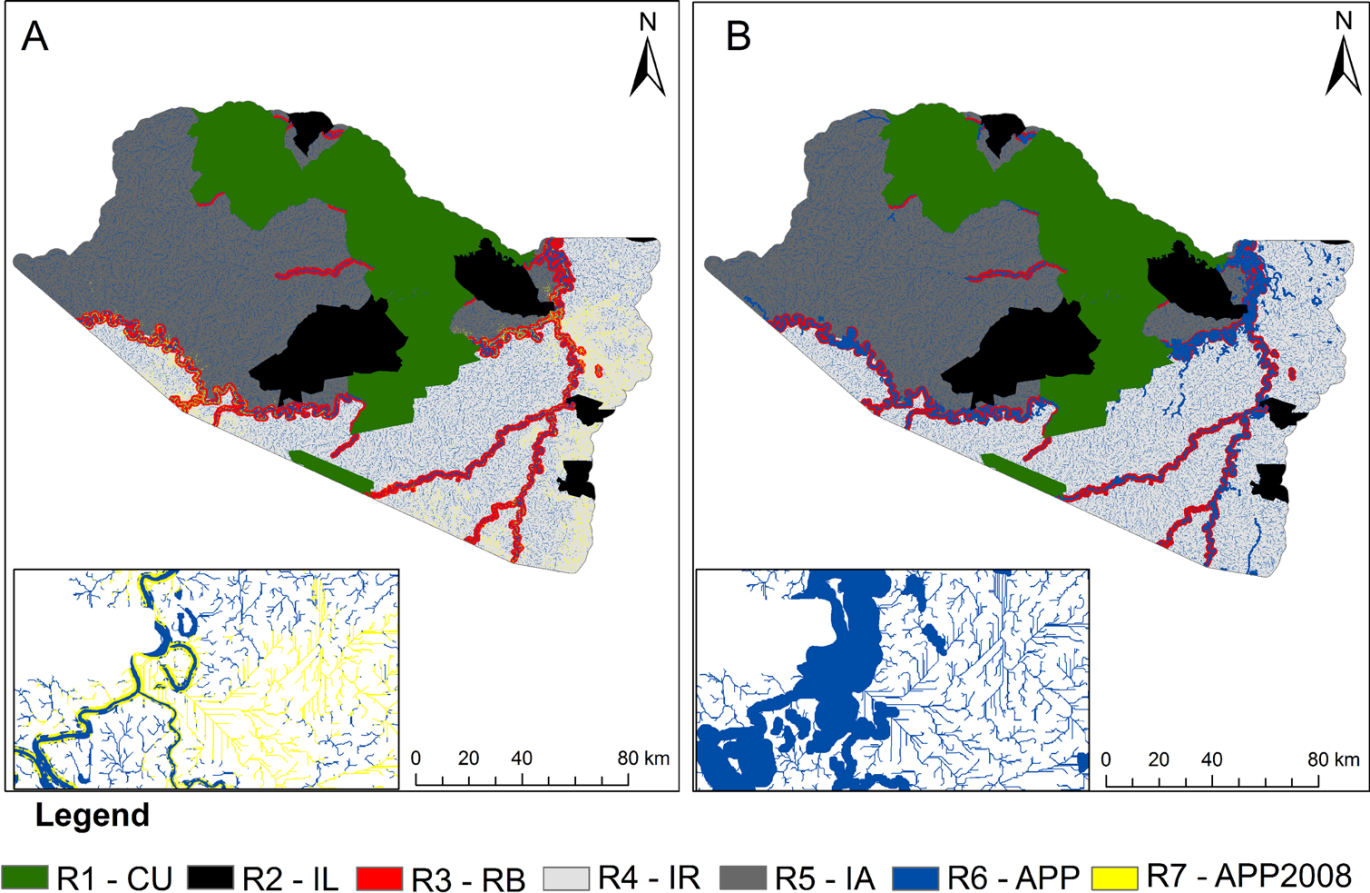
Map of regions for the scenarios with details for the regions for APP and areas of permanent protection cleared by 2008 (APP2008). **a** 2012 Forest Code (Law 12.651/2012), **b** 1965 Forest Code (Law 4.771/1965)

The spatial resolution used in the input maps was 30 m and the cartographic projection applied was UTM Zone 19 South and Datum WGS-1984. The inputs to the model were:

-*Map of static variables:* Vegetation (Brazilian Institute of Geography and Statistics: IBGE); soil (IBGE); altitude (SRTM); slope (derived from the altitude map); Watercourses (extracted from the land-cover map); roads (from the Remote Sensing Center, Federal University of Minas Gerais: CSR/UFMG) updated with the roads identified in satellite images (2005 and 2012) and on-site during fieldwork in 2012; CUs (from IBGE and the Amazonian Protection System: IBGE/SIPAM); and ILs (from the National Indian Foundation: FUNAI);

-*Maps of friction and attractiveness:* Created in Dinamica-EGO through multi-criteria analysis by assigning values (weights) to features that have a predisposition to either attract or repel the construction of roads and, consequently, speed or slow deforestation. Factors of attraction are roads and watercourses, while repulsive factors are ILs, CUs and areas with steep slopes (Soares-Filho et al. 2009);

- *Land-cover map:* For the year 2012 (Fig. 1);

- *Road map:* (CSR/UFMG) updated with roads identified in satellite images for 2005 (calibration phase) and 2012 (simulation phase) and on-site in 2012; this is necessary for the model’s “road-builder” module and for calculation of transition-probability maps and rates of deforestation within the program;

-*Map of regions:* This map compartmentalizes the study area into “regions” and projects deforestation in a different way for each region (Table 3 and Fig. 3). This considers the level of protection of the area, deforestation dynamics along rivers and roads, and APPs. The study area was divided into six regions in the “Baseline Scenario” and the “1965 Scenario” and into seven regions in the “2012 Scenario” (Table 3). For each region, the transition rates, weights of evidence, and dynamics of road construction were distinct.

**Table 3:** Regions used in the A-Eco model

Region	Description of the region
CU	Conservation units
IL	Indigenous lands
RB (River buffer)	One-km buffer around the rivers with width ≥30 m classified in the land-cover map. This region considers deforestation by the riverside dwellers (*ribeirinhos*) who inhabit the shores of navigable rivers
IR (Influence of roads)	The southern and southwestern portions of the municipality, which are under the influence of highways
IA (Isolated areas)	The northern and eastern portions of the municipality, which are isolated geographically with no access by land
APP	Areas of permanent preservation (APPs) on the banks of watercourses. This overrides all regions except CU and IR, since the Forest Code does not apply in the same way to these areas
APP2008	APP areas where deforestation took place before 2008. This region was only used for the scenario that considers Law 12,651/2012

Weights of evidence represent the susceptibility of a cell to changing from one state to another. For example, cells in the forest class that are located away from deforested areas or from roads are less susceptible to changing from forest to deforestation, since they have lower weights compared with forest cells located next to these areas. The transition rates represent the overall amount of change, i.e., they determine the number of cells that will undergo the transition in each iteration. The transitions used were:

- forest to deforestation;
- deforestation to secondary vegetation (regeneration);
- secondary vegetation to deforestation (cutting of secondary vegetation).

Deforestation rates were obtained according to the equation used by Yanai et al. (2012), where the rates are updated in each iteration in accord with the increment of roads in the model. Rates of cutting and regeneration of secondary vegetation were determined from the calculated transition matrix in Dinamica-EGO.

### Calibration and Validation

“Calibration” refers to the “estimation and adjustment of model parameters and constants to improve the agreement between model output and a data set,” while “validation” means that a model is “acceptable for its intended use because it meets specified performance requirements” (Rykiel 1996). In the process of calibration, weights of evidence and transition rates were determined using the 2005–2010 period.

Validation was carried out by applying the weights and rates found in the same study area for the period from 2005 to 2012. As input, the simulation used the land-use map for 2005 and ended by simulating the map for 2012, which was then compared to the map of real deforestation by that year (from PRODES: Brazil, INPE 2016) in an effort to achieve the maximum possible spatial similarity.

The weights and the rates were obtained and applied to each region. In the scenario for Law 12,651/2012, the sizes of the regions were changed and the weights and transition rates were therefore recalculated for this scenario.

**Fig. 4:**
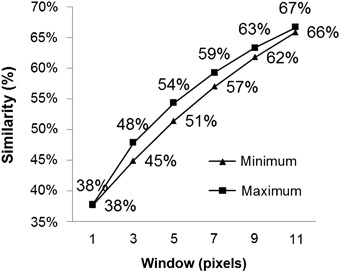
Percentage of similarity between the simulated and the real map for 2012

For the allocation of the land-cover classes, the model was validated spatially with 51% minimum similarity for a 5 × 5 pixel window (Fig. 4). For the quantitative validation, the difference between the real map and simulated map are given in Table 4.

**Table 4:** Quantitative validation the A-eco model applied to the study area

Class	Absolute difference (real map–simulated map) (km²)	Percentage difference (%)
Forest	2.475	0.33%
Deforestation	7.588	0.92%
Secondary vegetation	−10.057	−12.63%

The difference between the real and the simulated map in the validation step shows that the model underestimated the forest and deforestation classes and overestimated the secondary vegetation (Table 4). The overestimation of the amount of secondary vegetation can be attributed to the difference between the calibration period (2005 to 2010) and the 2012 map used for validation, since in 2012 there was 24% less secondary vegetation than in 2005 and 30 percent less than in 2010.

### Simulated Scenarios

Three deforestation scenarios were simulated from 2013 to 2025, using Dinamica-EGO software:

*-Baseline Scenario*: The transition rates consider the deforestation trend in recent years. There is no restriction on the use of APPs, a premise that is closest to the real situation, considering that only a few of the landholders respect the legislation in Boca do Acre. In this scenario, only six regions (Table 3) were considered and the APPs were based on Law 4771/1965.

-*Law 4771/1965 Scenario (1965 Scenario):* This is a scenario where forest legislation regarding APPs along the banks of watercourses (calculated on the basis of the maximum water level) was fully respected beginning from the first iteration (i.e., no deforestation occurs in these areas). This scenario assumes that starting to respect the Forest Code in private properties would stimulate “leakage,” where deforestation that would otherwise occur in the APPs moves elsewhere to areas of intact vegetation that are unprotected (public and non-designated forest areas) (Sparovek et al. 2012). All of the gross rate of deforestation and of cutting secondary vegetation in the APPs was transferred and recalculated in terms of the net rate for cutting secondary vegetation in adjacent areas. This made it possible to observe and compare the effect of the two legislations in the scenarios. The APP region in the 1965 Scenario was the same as that used in the Baseline Scenario.

-*Law 12,651/2012 Scenario (2012 Scenario)*: The APPs built for this scenario were based on the “regular” channel of each watercourse. In these areas, the Forest Code was fully respected from the first iteration, thus preventing deforestation in APPs. In addition, the “APP2008 region,” which refers to deforestation through 2008 in APPs, was added. Under Law 12,651/2012, these cleared areas are exempt from being fully recovered, and agricultural activities can be continued. Requirements for recovery of the vegetation are in accordance with the size of the property. The largest restoration is required for properties with areas greater than four tax modules (i.e., 4 × 100 ha in Boca do Acre), with the width of APP restoration being at least 20 m and the maximum requirement being 100 m. The transitions for cutting secondary vegetation were maintained in the simulation for the APP2008 region due to the spatial resolution used being 30 m and because it is assumed that the minimum required under the 2012 Forest Code will be adopted. Note that the APP2008 region was only used in the simulation of this scenario, this area being included in the APP region in all of the other analyses.

In all scenarios a mask was used to nullify values in urban areas in order to prevent regeneration in these areas, even when they were located on the banks of rivers. These sites have human occupation, impeding regeneration of the vegetation.

### Estimates of Carbon Stock Loss and Annual Carbon Emissions

Biomass values were obtained based on the forest type indicated at each location by the vegetation map of the IBGE (Brazil, IBGE 1992) and the dry mass value above ground and below ground for each forest type calculated by Nogueira et al. (2008a). For areas of forest with a predominance of bamboo, which is abundant in Boca do Acre (Nelson et al. 2006), we used the methodology presented by Vasconcelos et al. (2013). This methodology uses the values for biomass of trees and palms with diameter at breast height (measured 1.3 m above the ground or above any buttresses) greater than 5 cm (Nogueira et al. 2008b), and adds the values obtained from the biomass equations developed by Nelson et al. (1999) for bamboos and by Gehring et al. (2004) for lianas, applied to the inventory carried out in Acre by de Oliveira (2000). Finally, necromass values are added (Nogueira et al. 2008a), obtaining the total biomass for the forest type with predominance of bamboo.

To determine the loss of carbon stocks, biomass values above ground and below ground were multiplied by the average proportion of carbon in dry biomass as determined by da Silva (2007). This proportion is 0.485.

The calculation of annual emissions included the secondary vegetation biomass based on the mean biomass growth rate of secondary vegetation found in abandoned cattle pastures in the municipalities of Paragominas and Altamira, Pará (Fearnside and Guimarães 1996). The average age of secondary vegetation was considered to be 5 years (Almeida 2009). Carbon was considered to represent 45% of the dry biomass of secondary forest (da Silva 2007).

## Results

### Comparisons Among Scenarios

**Fig. 5:**
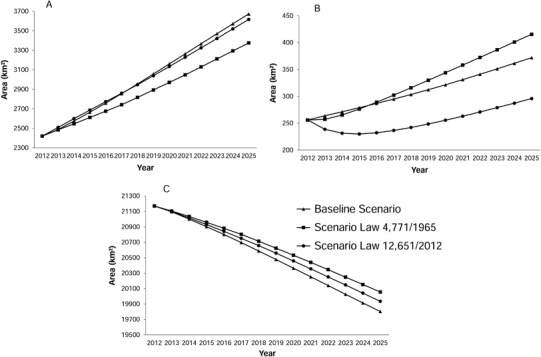
Temporal distribution (2012 to 2025) of land-cover classes based on simulated scenarios. **a** Deforestation, **b** Secondary vegetation, **c** Forest

The biggest difference between the scenarios occurred in the areas of secondary vegetation, where the 1965 Scenario was 10.4% higher than the Baseline Scenario and 20.5% higher than the 2012 Scenario (Fig. 5a). In the case of deforestation through 2025, the 2012 Scenario resulted in almost as much deforestation as the Baseline Scenario (3616 and 3672 km², respectively). The area deforested in the 1965 Scenario was 8.1% less than in the Baseline Scenario and 6.7% less than in the 2012 Scenario (Fig. 5b).

The Baseline Scenario had the greatest reduction in forest cover by 2025 (1368.6 km²) followed by the 2012 Scenario (1236.3 km²) and the 1965 Scenario (1115 km²). Average annual losses were 105.3 km² (Baseline Scenario), 95.1 km² (2012 Scenario), and 85.8 km² (1965 Scenario) (Fig. 5c).

Compared to the initial year of the modeling (2012), forest loss was 0.5% (121.30 km²) lower in the 1965 Scenario than in the 2012 Scenario, and the loss in the 1965 Scenario was 1.2% (253.64 km²) lower than in the Baseline Scenario. The increases in secondary vegetation of 45.4% (Baseline Scenario), 62.4% (1965 Scenario), and 15.6% (2012 Scenario) represent, respectively, 8.9, 12.3, and 3.1 km². The difference in the increase of the deforested areas exceeds 10% when the 1965 Scenario is compared to the other scenarios (297.01 km² relative to the Baseline Scenario and 240.93 km² relative to the 2012 Scenario) (Table 5).

**Table 5:** Difference between the scenarios in 2025 and the initial (2012) map

Class	Baseline scenario	1965 scenario	2012 scenario
Forest	−6.5%	−5.3%	−5.8%
Deforestation	51.8%	39.5%	49.4%
Secondary vegetation	45.4%	62.4%	15.6%

### Comparisons Among Regions

**Fig. 6:**
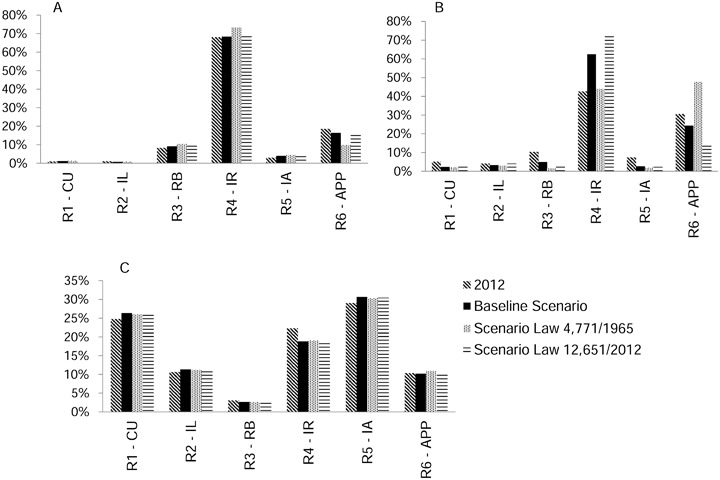
Distribution of land-use classes by region in 2012 and for each scenario in 2025. **a** Secondary vegetation, **b** Deforestation, **c** Forest. *R1-CU* Region 1 Conservation units, *R2-IL* Region 2 Indigenous lands, *R3-RB* Region 3 River buffer, *R4-IR* Region 4 Influence of roads, *R5-IA* Region 5 Isolated areas, *R6-APP* Region 6 Areas of permanent protection

In the initial year of the simulations (2012), 43% of the secondary vegetation was located in the area of IR (region). By 2025, the percentage of secondary vegetation in this region increased to 62% in the Baseline Scenario and 73% in the 2012 Scenario, while in the 1965 Scenario it remained stable at 44%. In the buffers along rivers (RBs) and in the isolated areas (IAs) there was a reduction in secondary vegetation in all scenarios as compared to 2012. In the APP region, there was an increase in secondary vegetation from 31% in 2012 to 48% in 2025 under the 1965 Scenario (Fig. 6a).

The percentage of deforestation remained stable in all regions for the Baseline Scenario and the 2012 Scenario. Only in the 1965 Scenario were there changes in distribution of deforested areas among regions, indicating increases of 2% river buffer (RB) and 5% (IR), and a reduction of 9% in the APP region (Fig. 6b).

There was an increase in forest cover in CUs, ILs, and IAs in all three simulated scenarios due to the absence of cutting secondary vegetation and, consequently, the steady growth of vegetation. However, in the IR region there was a reduction in the area of forest due to the IR (Fig. 6c).

### Estimates of Carbon Stocks and Emissions

**Fig. 7:**
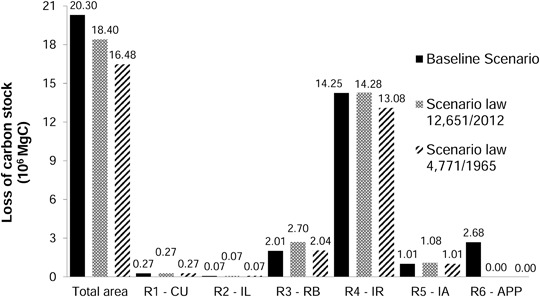
Losses of carbon stocks in 2025 for the study area a whole and by region. *R1-CU* Region 1 Conservation units, *R2-IL* Region 2 Indigenous lands, *R3-RB* Region 3 River buffer, *R4-IR* Region 4 Influence of roads, *R5-IA* Region 5 Isolated areas, *R6-APP* Region 6 Areas of permanent protection

The largest reduction in carbon stocks occurred in the Baseline Scenario, a reduction equivalent to 3.74% of the initial inventory (542.95 × 10^6^ MgC). The 1965 Scenario had the smallest reduction (3.03%), followed by the 2012 Scenario (3.39%). The smallest changes were observed in CUs and ILs, with virtually no difference between the three scenarios. The largest reductions in carbon stocks occurred in the region under the IR. In the APP region, reduction in carbon stocks only occurred in the Baseline Scenario (Fig. 7).

**Fig. 8:**
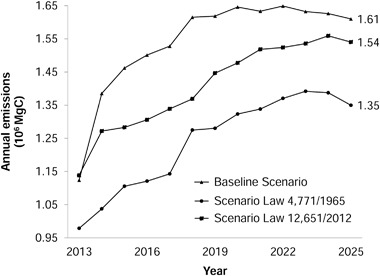
Comparison of annual emissions of carbon in each simulated scenario

Annual carbon emission in each scenario grew until approximately the sixth iteration (2019), after which it followed a constant pattern with a small decrease in the last few years. Again, the 1965 Scenario had the lowest emission, with a peak of 1.39 × 10^6^ MgC in 2023. The emission peaks for the Baseline Scenario and the 2012 Scenario were, respectively, 1.65 × 10^6^ MgC (2022) and 1.56 × 10^6^ MgC (2024) (Fig. 8).

## Discussion

### Performance of the A-Eco Model

The deforestation rate in Boca do Acre increased through 2010, stabilized in 2011 and 2012, followed by a decrease in 2013 and increases in 2014 and 2015 (Brazil, INPE 2016). At the same time, secondary vegetation cutting represented an increase in the use of abandoned or fallow areas between 2011 and 2012 that could have been influenced by the paving of Highway BR-317, which was restarted in 2011 as part of the federal government’s Second Program for the Acceleration Growth (PAC 2). Improved infrastructure created an incentive for the farmers and ranchers to return to use areas that were in the process of regeneration. Highway BR-317 from Rio Branco, Acre to Boca do Acre, Amazonas was completely paved in August 2012, except for the parts where the highway passes through ILs. In fact, the growth of deforestation in 2010 was already associated with the possibility of paving the highway (Piontekowski et al. 2011).

Even with the overestimate of secondary vegetation, our model can be considered good if compared with models in other studies (Table 6). For a margin of error of 150 m our model produces a minimum of 51% similarity.

**Table 6:** Validation of deforestation models built in Dinamica-EGO

Resolution	Validation (%)	Window (pixels)	Margin of error	Author
30 m	51	5 × 5	150 m	This study
250 m	23.1 to 73.8	1 × 1 and 11 × 11	–	Yanai et al. 2011
100 m	59	–	1 km	Teixeira and Soares-Filho 2009
500 m	54	5 × 5	–	Vitel 2009

### The Role of Respect for the Law

Forest loss expected in the Baseline Scenario (Fig. 6c) is due to continued violation of the law. In this scenario, forest cutting was governed only by rates and weights of evidence from recent years calculated for each region. A similar condition is expected if the new legislation is not accompanied by policies to encourage the reduction of deforestation and if the law is not better enforced than was the case under the previous Forest Code. By itself, the law is not able to change reality (Breda et al. 2011). One way to ensure the effectiveness of the Forest Code may be through public policies that value more sustainable methods of production and facilitate and promote oversight.

Although the 1965 Forest Code was widely disobeyed, it included two important instruments for protecting forests, water resources, soil, and biodiversity: APPs and legal reserves; these continue in the 2012 Forest Code.

Another aggravating factor for compliance with the Forest Code in Amazonia is the lack of delimitation of rural properties (Sparovek et al. 2011), which generates insecurity from risk of land invasion either by small squatters or by large “land grabbers” (*grileiros*). In recent years, the federal government’s Legal Land Program (*Programa Terra Legal*) has tried to carry out delimitation and regularization of land holdings, but so far without success in the municipality of Boca do Acre. Expectations for delimiting properties are now focused on the Rural Environmental Register (Cadastro Ambiental Rural (CAR)), which is a mechanism required by Law 12,651/2012. As a prerequisite for regularizing areas that were deforested illegally prior to July 2008, the CAR requires delimitation of each property and its respective APPs and legal reserve (80% of each property in Amazonia). The CAR consists of an electronic register that was conceived to assist environmental and economic planning, the control and monitoring of rural areas and the recovery of degraded areas (Laudares et al. 2014). Joining the CAR should have happened within 2 years after the enactment of the 2012 Forest Code (i.e., by 25 May 2014), but progress was modest by that date in Boca do Acre.

An unsuccessful attempt to achieve a similar delimitation of legal reserves and APPs was made through Decree 6514/2008, which required the recording of the legal reserve in the same period but did not offer a benefit (“amnesty”) to landholders, instead specifying punishment by fines for non-compliant properties. The result of the widespread non-compliance was that the government reissued the decree each year until the revised Forest Code was adopted in 2012.

An important problem caused by pardoning (“amnesty”) of illegal deforestation prior to 2008 is that it engenders the expectation that landowners can clear illegally and then later get the illegal deforestation “regularized” or “legalized.” “Amnesties” like this weaken public belief in the need to respect legislation, undermining the rule of law (Fearnside 2010). Ironically, proponents of the 2012 revision of the Forest Code argued that the previous Forest Code was widely disobeyed, whereas the revised Code would be respected, thereby making it a positive step for the environment. However, the “amnesty” included in the revision represents the seed of the very disrespect for law that the revision proponents claimed would end. Ever since its colonial past, Brazil has had a long tradition of having many laws “on the books” that are not enforced or obeyed, and the general assumption in many countries that what is written in law will be automatically translate into actual behavior does not apply (Rosenn 1971). This results in a continual testing of the limits of compliance with any new law and an ability to find ways of circumventing restrictions by informal means (the Brazilian “*jeito*”). This has profound consequences for efforts to control deforestation (Fearnside 1979, 2001).

### Secondary Vegetation and Deforestation Leakage

**Fig. 9:**
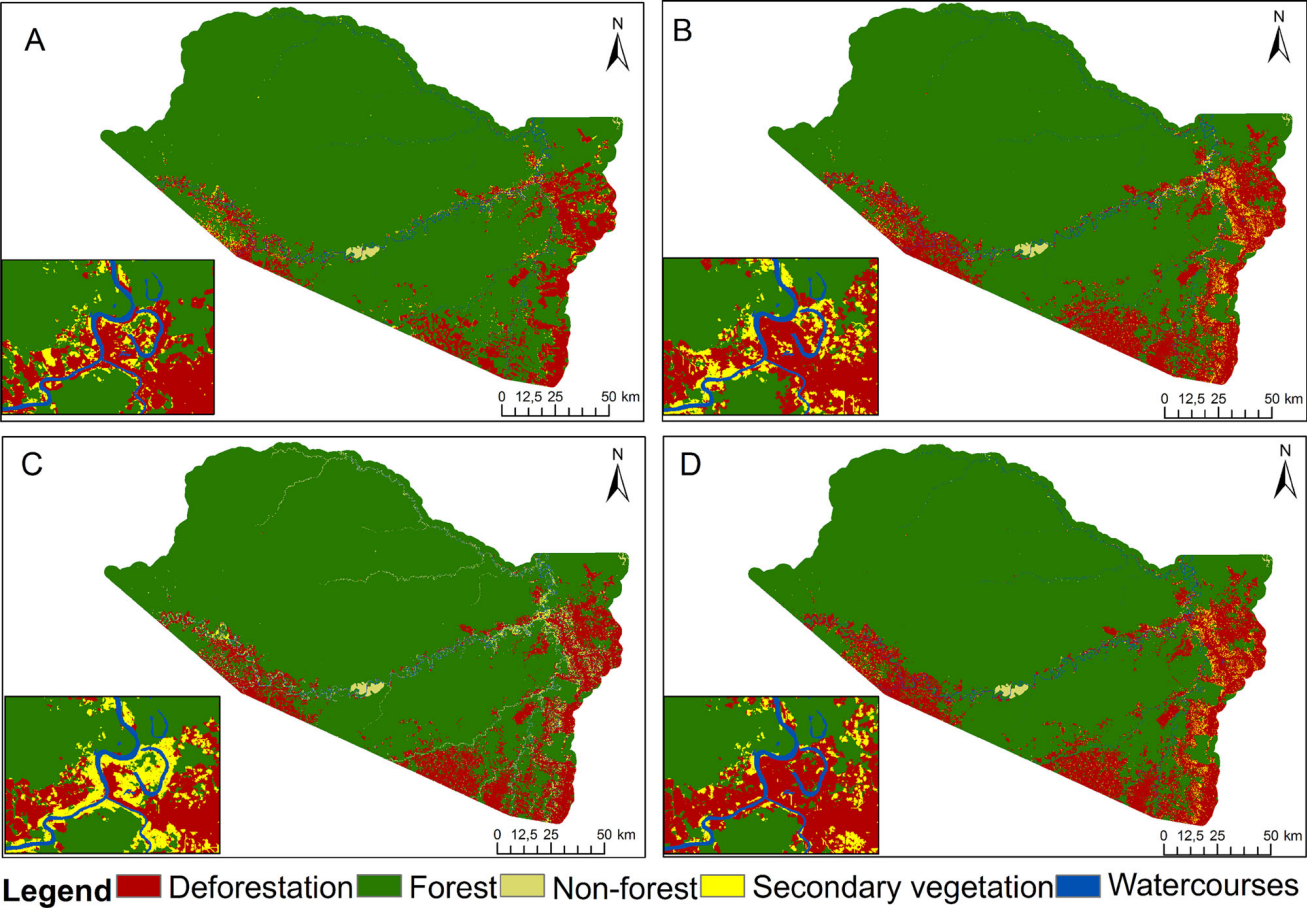
Initial map (2012) and modeled scenarios (2025). **a** Observed land use (2012), **b** Baseline scenario (2025), **c** 1965 Forest Code scenario (2025), **d** 2012 Forest Code scenario (2025)

There was a large difference between our simulated scenarios in relation to secondary vegetation (Figs 5b and 6a). This was associated with prohibition of cutting in the APP region. Despite displacement of deforestation from the APPs to other land categories in the form of cutting secondary vegetation, regeneration on the banks of watercourses was intense in the 1965 Scenario (Fig. 9). In the 2012 scenario, the situation was reversed because cutting secondary vegetation was permitted in the portions of the APPs with deforestation before 2008 (the “APP2008” region). This had a direct influence on the distribution of deforestation among the regions. In the 1965 Scenario, deforestation increased in the “RB” region and decreased in the “APP region” (Fig. 6b).

The value of this information is notable in light of the functions and importance of the APPs on the banks of watercourses. Maintenance of vegetation in riparian areas has social, economic, and ecological consequences even though this is predominantly secondary vegetation. Over the long term, the secondary vegetation will turn into forest. Additionally, much of the illegally cleared vegetation that will not be recovered under the 2012 law is located in wetlands that account for about 30% of Amazonia and provide environmental services such as groundwater recharge, regulation of biogeochemical cycles, and maintenance of carbon stocks. In addition, these areas serve as habitat for fauna and provide vital services for the human populations (Piedade et al. 2012). If the annual flood pulses become higher, the natural process of methane release by wetlands (Singh et al. 2000) would be intensified. Increased flood peaks are predicted in western Amazonia as a result of climate change (Zulkafli et al. 2016).

The secondary vegetation itself has an important role in absorbing greenhouse gases (Fearnside 1996), as can be seen in the last years of the simulation where there was a reduction in emissions (Fig. 8). This drop is a result of increased regeneration and, consequently, higher carbon absorption by the vegetation. Even with the increased deforestation and with the cutting rates for secondary vegetation imposed in the 1965 and 2012 Scenarios, emissions were, respectively, 0.04 × 10^6^ and 0.02 × 10^6^ MgC lower in 2025 as compared to 2024. The increasing importance of carbon uptake by secondary vegetation at the very end of the simulation would probably continue were a longer time period considered.

### Effects of Forest Protection

The largest deforestation and carbon stock losses were related to the IR, since the presence and the paving of highways are factors that are attractive to deforestation (e.g., Soares-Filho et al. 2004) (Fig. 6b). In the 1965 Scenario, 79% of the carbon emissions occurred in the IR region, while in the Baseline Scenario 70% of the emissions occurred in the APP region, and in the 2012 Scenario 77% occurred in the RB region (Fig. 9).

On the other hand, in the protected areas (CU and IL regions) and in the IAs, the proportion of forest increased (Fig. 6c), and the reduction in carbon stock was lower (Fig. 7). CUs and ILs have an important role in storing carbon and in blocking deforestation (e.g., Nepstad et al. 2006; Soares-Filho et al. 2010). Our model had no restriction on deforestation in protected areas, deforestation activity being governed by observed historical rates, which were low in these areas.

Comparing the three scenarios, it is evident that complying with the legislation is important for reducing deforestation and greenhouse-gas emissions. If the 1965 Forest Code had been supported by more efficient public policies and had been fully complied with, it would have further guaranteed the protection of the country’s forests, soils, biodiversity, and water resources. This, of course, is no longer a real option. Regarding the 2012 Code, there are still no arguments that can ensure that it will be respected.

### Policy Implications

In view of the results, when considering deforestation or emissions and protection of fragile areas, maintenance of Law 4771/1965 would have been the best option, provided that it were enforced. The scenario based on maintenance of activities without improvements in the implementation of laws had the worst prospects. Only changing the legislation does not imply the best performance, especially when it encourages impunity, as is expected to result from the “amnesty” that the new Forest Code granted for 43 years of violations under the previous Forest Code (Fearnside 2010; IPAM 2011; Roriz and Fearnside 2015). The law only serves as a reference for what should or should not be done. Endless repetition of claims that the 2012 Forest Code will be enforced and obeyed do not make this happen in practice.

The main policy implication of the present study’s results is that the Brazilian government needs to undertake a large-scale and immediate effort to enforce the current Forest Code. This is not what is happening in practice. Brazil’s commitment under the Climate Convention’s 2015 Paris Accords does not foresee eliminating “illegal deforestation” until 2030 (Brazil 2015, p. 3). A constitutional amendment (No. 95, formerly PEC-55) enacted in 2016 freezes the federal budget for the next 20 years, with only education and health (not environment) guaranteed a minimal level of support (Brazil 2016). The federal budget for the Ministry of Environment was cut by 43% in 2017 (Dasgupta 2017).

## Conclusion

The scenario with full protection of the APPs on the banks of watercourses under the 1965 Forest Code (Law 4771/1965) showed better results in curbing deforestation and in maintaining carbon stocks, in sequestering carbon and in mitigating climate change than did the 2012 Forest Code (Law 12,651/2012). However, if the 2012 Forest Code were fully enforced, it would result in lower rates of deforestation and emissions as compared to the Baseline Scenario (observed historical behavior without obeying either Forest Code). Within the parameters analyzed in this study, the greatest problem with the 2012 Forest Code is the weakening of protection of forest/river transition ecosystems, such as floodplains. The results indicate the need for concerted government action to enforce the current (2012) Forest Code.
